# Comparative Study of Endovascular Aneurysm Repair in Patients with Narrow Aortic Bifurcation Using the Unibody AFX2 vs the Bifurcated ALTO Endoluminal System

**DOI:** 10.3400/avd.oa.25-00027

**Published:** 2025-06-14

**Authors:** Nikolaos Kontopodis, Michalis Pesmatzoglou, Ifigeneia Tzartzalou, Konstantinos Litinas, George Tzouliadakis, Nikolaos Galanakis, Elias Kehagias, Christos Ioannou

**Affiliations:** 1 Vascular Surgery Department, Medical School, University of Crete, Heraklion, Greece; 2 Interventional Radiology Unit, Medical School, University of Crete, Heraklion, Greece

**Keywords:** EVAR, anatomy, aneurysm, eligibility, reinterventions

## Abstract

**Objectives:** A narrow aortic bifurcation poses technical challenges during endovascular aneurysm repair (EVAR). We aim to compare the unibody AFX2 (Endologix, Irvine, CA, USA) vs the bifurcated ALTO (Endologix) system in EVAR patients with a narrow bifurcation.

**Methods:** Retrospective single-center study, including patients undergoing standard EVAR over 3 years. Patients with a bifurcation diameter <20 mm were identified, and outcomes were compared between the AFX2 and ALTO groups. Primary endpoints were primary and overall technical success, primary and overall clinical success, rate of adverse limb events, rate of limb occlusion, and need for secondary interventions. The analysis was repeated using a diameter threshold of <18 mm.

**Results:** Among 151 cases, 26 presented with bifurcations <20 mm and 12 with <18 mm. In the primary analysis, 15 patients were treated with the AFX2 and 11 with the ALTO endograft. Both groups achieved 100% technical and clinical success. No limb occlusions occurred, and no reinterventions were recorded. Preoperative anatomy was suitable for ALTO in all cases, while eligibility for AFX2 was 15 out of 26 cases. Secondary analysis displayed similar results.

**Conclusions:** In patients with narrow aortic bifurcation, the AFX2 endograft can be safely used when appropriate anatomic conditions are met. If the AFX2 system is unsuitable due to other anatomic restrictions, the ALTO endograft is a viable alternative.

## Introduction

Endovascular abdominal aortic aneurysm repair (EVAR) currently represents the primary treatment modality for patients with abdominal aortic aneurysms (AAAs). Both in elective and emergent settings, current guidelines indicate that EVAR should be the preferred technique for AAA repair, in the presence of suitable anatomy.^1)^ Indeed, baseline anatomy is important for a technically successful procedure with a sustained outcome.^1)^ Anatomic suitability is mostly determined by taking into account the instructions for use (IFU) of each endograft, that is, if the anatomic variables of a given AAA fall within the IFU of the endoluminal system that is used.^2)^ Additionally, general anatomic criteria have been proposed to distinguish between friendly and hostile aortic anatomy, defining both strict and more liberal anatomic definitions.^3)^

The morphologic indices that are included in the IFU of endografts and are considered important to define anatomic suitability are aortic neck diameter, length and angulation, iliac artery size and length, and adequacy of access vessels. During EVAR planning, patients with a narrow aortic bifurcation are often encountered, and in such instances, there is always a concern that competition of 2 limbs inside this narrow space may result in complications, such as inability of the limb to fully expand, or even collapse and occlude. Remarkably, the size of the aortic bifurcation is not typically included in the IFUs of currently available endografts or in other anatomic definitions, so clinicians usually base treatment decisions on their experience and clinical judgment.^2,4)^

To avoid the possibility of graft collapse and occlusion in patients with a narrow aortic bifurcation, specific endografts such as the unibody AFX2 (Endologix, Irvine, CA, USA) system with anatomic fixation offer obvious advantages. The AFX2 system has a unique design that does not include the presence of limbs inside the aneurysm sac. On the contrary, the main body of the endograft sits on the aortic bifurcation, where it splits into the unibody’s integrated iliac limbs, sitting in the common iliac arteries.^5)^ For this reason, this endograft has previously been indicated as an ideal platform to address narrow aortic bifurcations.^6)^ On the other hand, there may be other morphologic characteristics of an AAA that preclude its use, such as short and angulated aortic necks or small access vessels, which may render other systems more suitable for implantation.

With the present study, we aim to compare outcomes in AAA patients with narrow aortic bifurcation treated with the AFX2 endograft versus those who, due to other adverse anatomic variables despite having a narrow bifurcation, were treated with a different, standard bifurcated endograft.

## Materials and Methods

### Study design and study population

This is a single-center retrospective observational study including patients with a narrow aortic bifurcation who underwent EVAR during a 36-month period, from April 2021 to March 2024. For primary analysis, narrow aortic bifurcation was defined based on a diameter threshold of <20 mm. Analysis was repeated taking into account a diameter threshold of <18 mm. Measurements were made on computed tomography (CT) images after 3-dimensional reconstruction in a plane perpendicular to the centerline, according to the methodology proposed in the Society for Vascular Surgery (SVS) guidelines.^7)^

No patients were excluded based on the type of endograft that was used. In the analysis, only patients undergoing standard EVAR for infrarenal AAA were included, while those subjected to endovascular repair of more proximal aortic pathologies, such as pararenal, suprarenal, or thoracoabdominal aortic aneurysm with fenestrated or branched endografts or parallel grafts, were excluded.

Routinely, we prefer to use the unibody AFX2 endograft for patients with narrow aortic bifurcation if the rest of the lesion’s anatomic features fall within the anatomic requirements of this system as defined in its IFU. This is because its unibody design avoids the risk of limb occlusion if both limbs are competing inside a narrow aortic bifurcation.^5)^ In case of adverse anatomic characteristics an alternative endograft was chosen to treat patients in an on-label fashion. The ALTO (Endologix) system has previously been shown to accommodate a wider range of anatomies within its specifications, compared to other contemporary endografts, and this represented our alternative if the AFX2 system was not indicated.^8)^ Specifically, an aortic neck length <15 mm, access vessel diameter <6.3 mm, and common iliac artery diameter >23 mm were considered as exclusion criteria for treatment with the AFX2 system.

The current analysis was performed and reported according to the Strengthening the Reporting of Observational Studies in Epidemiology (STROBE) reporting standards for observational studies.^9)^ The Institutional Review Board provided approval for the conduction of this study (IRB number: 820/20/29.10.14).

### Implantation technique

In cases where the ALTO system was used, some modifications of the usual technique were undertaken to facilitate successful implantation. Specifically, while during the standard implantation technique, the contralateral limb is cannulated and deployed and, after that, the ipsilateral limb is inserted, in cases with a narrow aortic bifurcation, the contralateral limb is inserted but is not deployed until the ipsilateral limb is inserted as well. When both limbs have been advanced into the intended position, they are deployed simultaneously by 2 operators. This is followed by aggressive and simultaneous ballooning of the limbs, aiming to achieve a 10-mm expansion for each limb.

The AFX2 system was deployed using the standard technique, where an integrated contralateral 0.014-inch guidewire is advanced from the ipsilateral side along with the unibody system. The floppy end of the crossover wire is then snared from the contralateral side and withdrawn. After insertion of the endograft from the ipsilateral side, the inner core and the contralateral wire are gently pulled down simultaneously for the endograft to sit on the aortic bifurcation. Then the main body and the limbs are deployed. Usually, a proximal endograft extension is deployed just caudal to the level of the renal arteries.

### Endpoints

Primary endpoints were primary and overall technical success, primary and overall clinical success, rate of adverse limb events, rate of limb occlusion, and need for secondary interventions. Secondary endpoints were various procedural parameters, such as procedural time, contrast volume, percutaneous access, blood loss, and length of hospital stay. These outcomes were reported according to the SVS Reporting Standards for EVAR.^7)^

### Statistical analysis

Continuous variables are reported as median and range. Differences between groups were compared using the Mann–Whitney U-test. Categorical variables are presented as count and percentage. Statistical significance was defined as a P-value <0.05, and all statistical analyses were performed using the Statistical Package for the Social Sciences v21.0 (IBM, Chicago, IL, USA) software.

## Results

### Demographics and baseline anatomy

During the study period, a total of 151 patients were treated with standard EVAR. Twenty-six patients, representing 17% of the study population, presented with a narrow aortic bifurcation. The median age of patients was 71 (range 52–88) years, while the vast majority were male (149/151 cases), as summarized in **Table 1**. Most of the included patients were treated with the ALTO endograft (n = 96), followed by the AFX2 endograft (n = 24), whereas in 31 cases, other systems were used, including Incraft-Cordis, Excluder-Gore, Percutec-Lombard, Aorfix-Lombard, Anaconda-Terumo, Altura-Lombard, and Treo-Bolton.

**Table:** Table 1 Demographic characteristics of the study population and type of endografts that were used

	ALTO	AFX-2	Other
No. of pts (total/narrow Bif)	96/11	24/15	31/0
Gender* (male/female)	11/0	15/1	-
Age*	72 (55–88)	73 (57–88)	-

*The values reported refer to the subgroup of patients with narrow aortic bifurcation.

Bif: bifurcation; pts: patients

For the primary analysis, among 26 patients with a narrow aortic bifurcation (<20 mm), in 15 cases the AFX2 system was used, while 11 patients were treated with the ALTO endograft. The median aortic bifurcation diameter was 18.4 mm (range 11.5–20 mm) among patients treated with the AFX2 and 18.5 mm (range 9.4–20 mm) in the ALTO group, with a nonstatistically significant difference between groups. Other anatomical variables such as AAA maximum diameter, neck diameter, and neck angulation were similar in both groups. Access vessel diameter was smaller among patients treated with the ALTO system, but this difference was not statistically significant. Neck length was significantly shorter among patients treated with the ALTO system. A summary of the anatomic variables of patients is provided in **Table 2**.

**Table:** Table 2 Baseline anatomic characteristics of the patients that were included

	ALTO	AFX-2	P-value
D-bifurcation (mm)	18.5 (9.4–20)	18.4 (11.5–20)	0.5164
Neck length (mm)	15 (7–37)	27 (14–46)	0.0485
Neck angle	24.5 (14–49)	25 (18–42)	0.9509
Neck diameter (mm)	21.4 (16.1–25.1)	22.7 (18–28.6)	0.6220
Access vessels minimum diameter (mm)	5.6 (3.2–11.4)	6.5 (5.1–9.5)	0.13
Dmax (mm)	60 (40–90)	57 (44–74)	0.68

For the secondary analysis, among 12 patients with a bifurcation diameter <18 mm, in 7 cases the AFX2 system was used and in 5 patients the ALTO endograft was chosen. The median aortic bifurcation diameter was 16.1 mm (range 11.5–18 mm) among patients treated with the AFX2 and 15.9 mm (range 9.4–17.8 mm) in the ALTO group, with a nonstatistically significant difference between groups. Again, the other anatomical variables were similar in both groups, except for neck length, which was significantly shorter among patients treated with the ALTO system (23 vs. 13 mm, P = 0.013).

### Primary endpoints

Regarding patients with a bifurcation diameter <20 mm, primary technical success was achieved in all but 1 patient in the ALTO group and 2 patients in the AFX2 group. In these latter 2 cases, an external iliac artery stenosis required additional stenting to restore lumen patency, which resulted in an overall technical success rate of 100%. One patient undergoing EVAR with ALTO presented with a Type Ia endoleak, which did not resolve with additional ballooning of the proximal part of the endograft, resulting in the single technical failure of this cohort. Nevertheless, this endoleak presented spontaneous resolution, and adequate sealing was observed during the 1-month follow-up. Clinical success was achieved in all patients, since no additional adverse limb events and/or need for reinterventions was recorded during a mean follow-up of 18 months. Notably, zero limb occlusions were observed in both groups. Two Type II endoleaks were recorded in each group, none of which was related to sac expansion. **Table 3** summarizes the primary endpoints of the analysis.

**Table:** Table 3 Summary of primary endpoints

	ALTO	AFX-2	P-value
Primary technical success	10/11	13/15	1
Overall technical success	10/11	15/15	0.42
Primary clinical success	11/11	15/15	1
Overall clinical success	11/11	15/15	1
Major adverse limb events	0	0	1
Limb occlusion	0	0	1
Reintervention	0	0	1

Taking into account patients with a bifurcation diameter <18 mm, technical and clinical success was achieved in all patients treated with either endograft.

### Secondary endpoints

Treatment with the AFX2 was associated with a significantly reduced iodinated contrast volume required during the primary procedure. The rest of the secondary endpoints were similar between groups. In the current cohort of patients, no ICU stay was required; all procedures were performed under regional anesthesia with a predominantly percutaneous approach. The median procedural duration was 77 minutes. The median length of hospital stay was 2 days. One patient in the ALTO group presented with a mild post-EVAR syndrome, which prolonged the hospital stay by 1 day, but did not result in any other complication. Secondary endpoints are summarized in **Table 4**.

**Table:** Table 4 Summary of secondary endpoints

	ALTO	AFX-2	P-value
Procedural time (min)	87 (60–140)	70 (45–120)	0.2875
Percutaneous access	11	12	0.49
Iv contrast (mL)	135 (70–160)	80 (40–120)	0.0161
Blood loss (mL)	300 (200–500)	300 (100–500)	0.4281
Length of stay (days)	2 (2–3)	2 (1–3)	0.3083
Post-EVAR syndrome	1/11	0/15	0.42

IV: intravenous; EVAR: endovascular aneurysm repair

Repetition of the analysis, taking into account patients with a bifurcation diameter <18 mm, did not indicate any significant differences in secondary outcomes between groups, except for contrast volume, which again was significantly lower in the AFX2 group (140 vs. 90 mL, P-value = 0.023).

### Baseline anatomy versus IFU requirements of endografts

Among 26 patients with a narrow bifurcation included in the primary analysis, all patients could be treated in an on-label fashion with the ALTO system, while only 15/26 could be treated with the AFX2 endograft. In other words, in all patients treated with the AFX2 system, if the aortic bifurcation diameter is excluded, preoperative anatomy would fall within the IFU of the ALTO system in all 15 cases. Conversely, those patients who were treated with the ALTO system could not be treated in an on-label fashion with the AFX2 endograft in all 11 cases due to anatomic restrictions regarding aortic neck length and/or narrow access vessels. Specifically, 5 patients presented with short aortic necks, 3 patients had small access vessels, and 3 patients had both short necks and narrow access. Regarding the secondary analysis, including patients with a bifurcation diameter <18 mm, again all 12 patients could be treated in an on-label fashion with the ALTO system, while the 5 patients who were treated with the ALTO system would fall outside the IFU of the AFX2 system.

## Discussion

**Figure 1:**
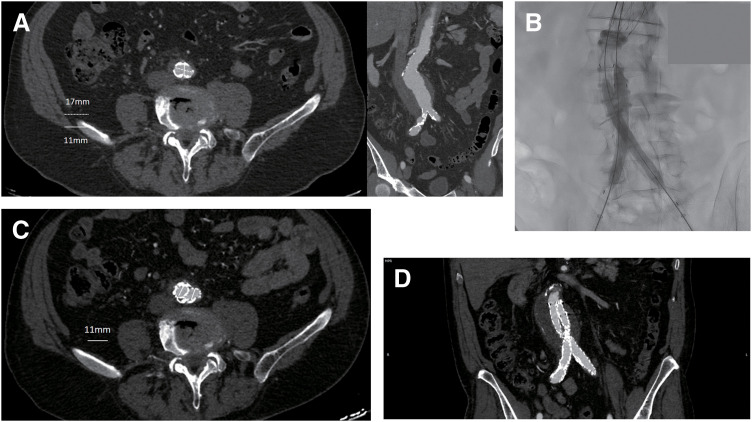
Fig. 1 (**A**) Axial and coronal views of a preoperative CT of a patient with a narrow aortic bifurcation. (**B**) Intraoperative image of simultaneous deployment of iliac limbs and kissing ballooning. (**C**) Postoperative CT image indicating successful expansion of iliac limbs at the level of the aortic bifurcation. (**D**) Postoperative coronal view indicating successful deployment of the limbs. CT: computed tomography

The current study examined the effect of the type of endograft on the outcomes of EVAR in patients with a narrow aortic bifurcation. Specifically, the outcomes of an endograft that by design seems more suitable for use in such a setting (AFX2) are compared with a system with a traditional bifurcated design (ALTO). Remarkably, both short- and mid-term results are similar between groups, with no limb-related adverse events recorded in any of the patients included in the analysis. This means that in our small study population, the ALTO system could be safely used in patients with narrow aortic bifurcations, without any compromise in the successful deployment and patency of iliac limbs. Of course, some technical modifications of the implantation technique are necessary in this setting. Specifically, the deployment of both limbs is performed simultaneously, followed by aggressive kissing ballooning at the level of the aortic bifurcation (**Fig. 1**). Remarkably, bifurcations as small as 10 mm were treated with the use of this technique.^10)^

A narrow bifurcation has previously been shown to be an adverse anatomic characteristic that can pose specific technical challenges and increase the potential for complications and need for reinterventions following EVAR. Previous studies have indicated that limb occlusion is usually reported at a rate around 3%–4%, although higher values have also been reported.^11–13)^ In the presence of such a complication, both surgical and/or endovascular therapies can be applied, but a potential for limb loss or even death cannot be excluded.^13)^ A narrow aortic bifurcation can predispose to iliac limb occlusion according to several studies.^14–17)^ Moreover, a recent meta-analysis summarizing comparative data between patients with a narrow and those with a standard aortic bifurcation, including 412 and 2261 subjects, respectively, indicated that limb stenosis and kinking were significantly more common among patients with a narrow bifurcation. This resulted in a significantly higher rate of adjunctive manipulations during the primary procedure, such as kissing balloon angioplasty, kissing stenting, or unilateral stenting to correct possible problems. The 30-day reintervention rate and limb occlusion rate were not significantly different between groups.^18)^

The AFX2 system has previously been shown to be an ideal platform to address narrow aortic bifurcations, which may be anticipated taking into account the unique design of this system—not including iliac limbs, but employing a unibody design with the main graft sited on the aortic bifurcation. Specifically, previous reports have indicated a universal technical success in patients with narrow distal aortas, with a very low reintervention rate due to limb stenosis or occlusion.^6,19,20)^ Other advantages, such as reduced procedural times and reduced contrast volume, have also been demonstrated for EVAR with the AFX system.^21)^ On the other hand, there have been some concerns regarding EVAR durability after implantation of the AFX system, mainly due to disconnection of the endograft’s components and the development of Type III endoleaks, but this mainly regarded previous generation of this platform and outdated IFUs.^5,22)^

One could argue that using the AFX2 unibody system in all standard EVAR patients presenting with a narrow aortic bifurcation would be a reasonable choice. Of course, this consideration ignores the other anatomic characteristics of AAAs, such as the proximal neck, distal landing zones, and access vessels. Indeed, specific anatomic characteristics of a given AAA may render an alternative endograft more suitable. The AFX2 system requires a neck length >15 mm, a neck angulation <60°, a distal landing zone with a diameter of ≥10 mm and ≤23 mm, and an iliac angle of ≤90° to the aortic bifurcation. Moreover, an access vessel larger than 6.3 mm is required.^8)^ Taking into account the above-mentioned anatomic specifications, a previous study recorded a 51% eligibility rate for AFX2 among 307 patients undergoing AAA repair (either open or endovascular) and 64% among 225 patients undergoing EVAR. The same study indicated that among most of the currently used endografts, the ALTO system presented the highest eligibility rates, with a value of 70% among the total study cohort and 89% among patients treated with EVAR.^8)^ According to these results, it is possible that a proportion of patients undergoing EVAR will fall outside the anatomic requirements of AFX2, but inside the IFU of other systems such as ALTO. In this instance, and in the presence of a narrow aortic bifurcation, a therapeutic dilemma regarding the choice of the most appropriate endograft arises. The results of the current analysis suggest that the ALTO endograft can be safely used in this setting. Following the above-mentioned arguments, the ALTO system was been chosen as the alternative to the AFX2 endograft for the treatment of patients with a narrow aortic bifurcation. Of course, other types of endografts could also be used in this setting, but in our experience, the wide range of anatomies that fall within the ALTO’s IFU, as well as its ultra-low profile, which makes it suitable to navigate through small and tortuous access vessels often encountered in patients with narrow bifurcations, make ALTO a reasonable choice for these cases.

Additionally, previous research has highlighted the fact that EVAR performed in an on-label fashion produces favorable outcomes compared to an off-label treatment. Specifically, a previous systematic review recorded a relative risk of 4.5 to develop a Type Ia endoleak in hostile versus friendly aortic neck anatomy, while aneurysm-related mortality was 9 times greater among patients in the former group.^23)^ Moreover, a recent systematic review comparing outcomes of EVAR inside versus outside the IFU recorded a significantly higher all-cause mortality among patients with a hostile baseline aortic anatomy.^24)^ Finally, a recent report including >15000 patients from the Vascular Quality Initiative confirmed these findings, highlighting the fact that neck characteristics outside of the IFU are independently associated with completion Type Ia endoleaks, perioperative mortality, 1-year sac expansion, and 1-year reinterventions among patients undergoing elective EVAR. These authors concluded that a continued effort is needed to improve the proximal seal in patients with neck characteristics outside the IFU undergoing EVAR.^25)^

Previous studies have used variable diameter thresholds to define a narrow aortic bifurcation, such as 16, 18, and 20 mm.^18)^ In the current study, we used both 20 and 18 mm values and performed the analysis for both cutoffs, finding similar results. If we had used only the 18-mm threshold, only 12 patients would have been included in the study group, which would limit the applicability of our results. Therefore, by using both thresholds, we were able to include 26 patients in the primary analysis, also confirming our findings in the subgroup of patients with a more strict definition for a narrow bifurcation.

The findings of the current study should be interpreted in light of some limitations. The main limitation is the small number of patients included in the analysis, which may limit the applicability of the present results. Additionally, the retrospective collection of data may be subject to information and recall bias.

## Conclusion

Narrow aortic bifurcation can be encountered in a significant proportion of patients undergoing EVAR. The unibody AFX2 system can be safely implanted in this setting in patients presenting a suitable anatomy for this specific endograft. In the presence of adverse anatomic characteristics for the AFX2 system, the ALTO system can be safely used in eligible patients with an AAA and a narrow aortic bifurcation.

## Declarations

### Funding

No funding was received for this study.

### Disclosure statement

The authors have nothing to disclose.

### Author contributions

NK: Conception, study design, and writing the manuscript

MP: Conception, study design, and writing the manuscript

IT: Data collection, literature review, interpretation of findings, and writing the manuscript

KL: Data collection, literature review, interpretation of findings, and writing the manuscript

GT: Data collection and statistical analysis

NG: Statistical analysis and interpretation of findings

EK: Study design and interpretation of findings

CI: Conception and overall responsibility

All authors: Critical review and revision, final approval of the article, and accountability for all aspects of the work.
